# Successful implementation of preventive measures leads to low relevance of SARS‐CoV‐2 in liver transplant patients: Observations from a German outpatient department

**DOI:** 10.1111/tid.13363

**Published:** 2020-06-22

**Authors:** Ramin Raul Ossami Saidy, Brigitta Globke, Johann Pratschke, Wenzel Schoening, Dennis Eurich

**Affiliations:** ^1^ Department of Surgery Charité – Universitätsmedizin Berlin Germany

**Keywords:** clinical research, epidemiology, liver transplantation, sars‐cov‐2

## Abstract

**Background:**

Immunosuppressed liver transplant (LT) patients are considered to be at high risk for any kind of infection. What the outbreak of the novel severe acute respiratory syndrome coronavirus 2 (SARS‐CoV‐2) means for the transplant cohort is a question that, as of now, cannot easily be answered. Data on prevalence, relevance of the novel virus, and clinical course of the infection in stable LT patients are limited.

**Methods:**

Nasopharyngeal swabs were performed in our outpatient department during the shutdown between March and April 2020 in Germany.

**Results:**

The prevalence of SARS‐CoV‐2 was 3%. Three out of a cohort of 101 LT patients were asymptomatic for respiratory diseases. Respiratory complaints were common and not associated with SARS‐CoV‐2 infection. The overall monthly mortality rate was 0.22% and did not show alterations during the shutdown in Germany.

**Conclusions:**

If preventive measures are applied, LT patients do not seem to be at a higher risk for SARS‐CoV‐2 infection. Telemedicine in the outpatient setting may help to maintain distance and to reduce direct patient contact. However, standard of care must be guaranteed for patients with relevant comorbidities in spite of pandemics, because complications may arise from preexisting conditions.

**Abbreviations**
ARDS: acute respiratory distress syndrome
CNI: calcineurin Inhibitor
COVID‐19: coronavirus disease 2019
LT: liver transplantation
MMF: mycophenolate mofetil
mTor: mechanistic target of rapamycin
SARS: severe acute respiratory syndrome
SARS‐CoV‐2: severe acute respiratory syndrome coronavirus‐2

## INTRODUCTION

Infection with the novel coronavirus (SARS‐CoV‐2) has the potential to lead to acute severe respiratory distress syndrome (ARDS) and is a tremendous health problem of pandemic dimension. Since the outbreak in China in December 2019, the official number of patients infected with SARS‐CoV‐2 has risen to almost 2.5 million worldwide and to almost 150 000 in Germany and counting, resulting in over 160 000 deaths globally and more than 4500 in Germany by the end of April 2020.^1^ There are different epidemiologic approaches to face the pandemic.^2^, ^3^ Most of them include social distancing, strict hygiene, imposition of quarantine in case of infection, and symptomatic treatment of ARDS ranging from oxygen application to extracorporal membrane oxygenation. Incidence and mortality of the viral infection differs largely among the countries, and clinical courses presented in the literature are more than alarming especially in high‐risk groups such as older patients, patients with malignant diseases, diabetes, hypertension, and assumedly on immunosuppression.^4^, ^5^

As previously hypothesized, immunosuppressed patients may be protected via their therapeutic regimen by putative attenuation of an overwhelming inflammatory response, for example, “cytokine storm” in case of initial infection with SARS‐CoV‐2, as this immune reaction is thought to contribute to ARDS in a significant manner.^6^, ^7^, ^8^

Overall affection of the liver in the COVID‐19 (coronavirus disease) pandemic seems to be mild, even in severe cases. However, elevated liver enzymes and bilirubin have been reported.^9^, ^10^, ^11^, ^12^ Their relevance for the clinical course remains unclear, and liver damage may be secondary as part of multi‐organ failure. A study of four deceased patients suffering from severe COVID‐19 showed none or only mild inflammatory aspects of the liver in post‐mortem biopsies.^13^ Still, a case report describes bystander‐hepatitis in a recently liver transplanted patient with a mild SARS‐CoV‐2 infection.^14^

Liver transplant (LT) patients are formally considered a vulnerable population, prone to all kinds of infections, because of the immunosuppressive medication. There are not enough data on the role of SARS‐CoV‐2 in stable liver transplant recipients. Therefore, we attempted to deliver some real‐world data from a high‐volume liver transplant outpatient clinic at the Charité Universitätsmedizin‐Berlin, Germany.

## 1 METHODS

**Figure 1:**
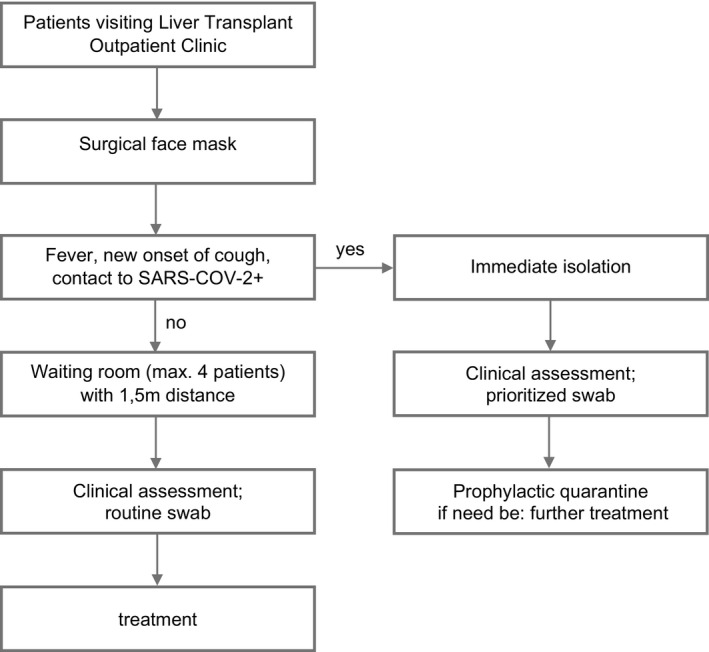
Established workflow in the LT outpatient clinic during the pandemic. Procedures for outpatients were established to ensure adequate hygienic prophylaxis with regard to the SARS‐CoV‐2 pandemic. Additional medical staff was recruited to facilitate these procedures, and patients were instructed to maintain strict measures

Our LT outpatient clinic currently cares for about 1500 patients Figure 1. The routine activities comprise planed check‐ups, routine blood test, and presentations for acute medical problems of any kind. As of March 2020, as the nationwide COVID‐19 lockdown commenced, we reduced our routine activities by 50%‐75%, in order to prevent a spread of infections among our immunocompromised patients. Still, regular care was provided for those in need of specialized treatment. Between the 23 of March and the 23 of April 2020, outpatients were specifically questioned for laryngopharyngeal or tracheobronchial symptoms, suggestive for COVID‐19 (ie, cough, fever, sore throat, dyspnea, new/changed sputum, new fatigue, exhaustion, new onset of diarrhea) and examined accordingly. Nasopharyngeal swabs were performed in all liver transplant patients during this period to detect SARS‐CoV‐2 disregarding the reason for the appointment and the complaints of the patients. Qualitative real‐time PCR was used to detect RNA of SARS‐CoV‐2. In this method, targeted RNA molecules are amplified and marked with fluorescence, thus assessing detectability, only, without quantitative analysis. The method proofed highly sensitive and specific if performed correctly.^15^, ^16^ The results were correlated with demographic and clinical data. The quarterly mortality between January 2019 and April 2020 was assessed in order to detect any previously unrecognized disturbances of this endpoint. Mortality was defined as number of deceased patients per month per total number of LT patients being followed up. Descriptive and statistical analysis was performed using SPSS software (IBM SPSS Statistics V.26). The data were acquired accompanying clinical routine, and the evaluation was performed retrospectively according to the Professional Code of the German Medical Association (article B.III.§15) based on the World Medical Association´s Declaration of Helsinki.

Patients that needed an urgent consultation in our transplant clinic were provided with a facemask upon arrival and immediately questioned for commonly known suggestive symptoms for COVID‐19, that is, fever, cough, and contact to infected people. If nothing applied patients were instructed to disinfect their hands and continued to the waiting area, reduced to a quantity of 4 seats with a distance of 1.5 m in between. Patients received a routine nasopharyngeal swab and treatment commenced as needed. If any of the aforementioned points applied, patients were immediately isolated received a clinical assessment and a prioritized swap. Patients went to prophylactic quarantine and received the respective treatment.

## 2 RESULTS

In 1 month during the SARS‐CoV‐2 pandemic, 101 LT patients received nasopharyngeal swabs at time of consultation. 55 (54.5%) patients were male and 46 (45.5) female, with a median age of 64.0 (19.8‐86.8) years. The underlying disease in the majority of patients was alcoholic (18.8%), HBV‐ or HCV‐associated (31.7%), and autoimmune liver disease (16.8%), including autoimmune hepatitis (AIH), primary biliary cholangitis (PBC), and primary sclerosing cholangitis (PSC). The backbone of immunosuppression was calcineurin inhibitors (mainly tacrolimus) in 80 (79.2%) cases individually combined with either mycophenolate mofetil or everolimus. 30 (29.7%) patients received tacrolimus monoprophylaxis. Three patients (3%) received combination therapy with corticosteroids as part of an individualized immunosuppression with 5‐20 mg/d. 9 (8.9%) patients were off immunosuppression for toxicity reasons for at least 6 months pertaining a stable graft function, except for one patient who was being weaned for 2 months. The extent of the immunosuppressive medication was highly individual, ranging from the standard tacrolimus trough level of 5‐8 ng/mL in immunologically unremarkable LT recipients in the first year, to individually adjusting and minimizing immunosuppression to very low‐level long term including weaning.

A vast majority of LT patients (n = 81.2%) had at least one of following comorbidities: obesity, defined as body mass index (BMI) ≥30 kg/cm^2^; n = 16, cardiovascular pathologies (n = 52) including coronary heart disease (n = 18), structural pulmonary disease (n = 27), active smoking (n = 14), diabetes mellitus (n = 25), current or recent (less than 6 months) neoplastic disease (n = 16), inflammatory bowel disease (n = 10) and advanced kidney disease with an estimated glomerular filtration rate (eGFR) of <45 mL/min (n = 17).

**Figure 2:**
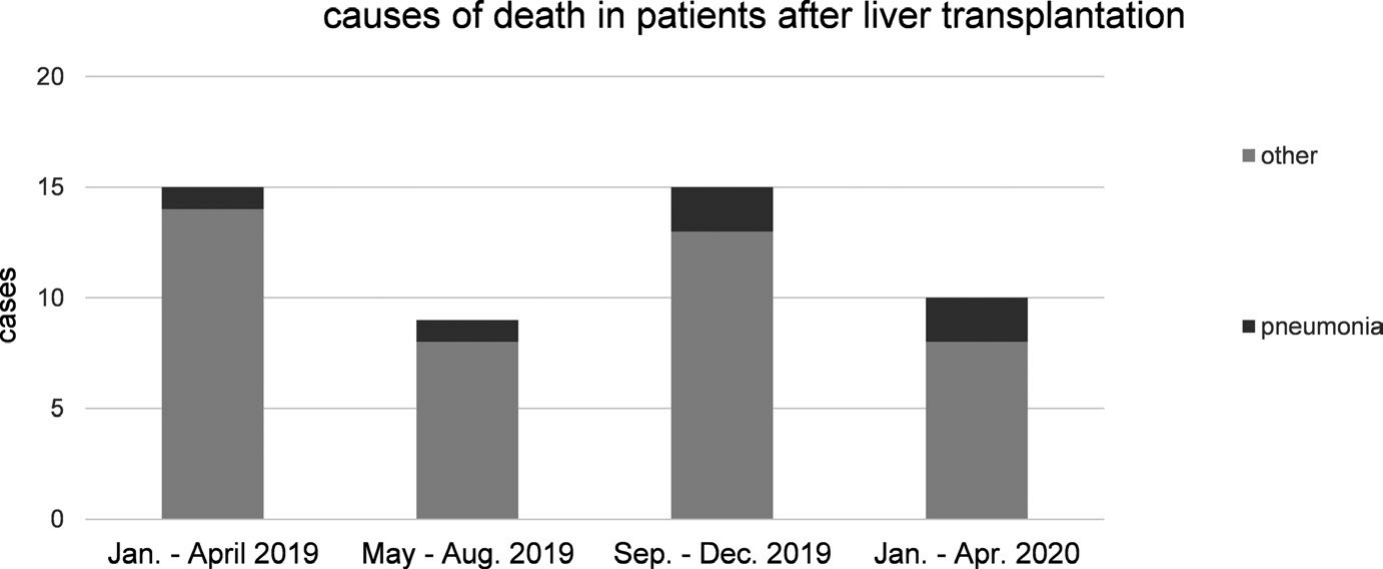
Pneumonia‐associated mortality 2019 including first 4 mo of 2020. Comparing the incidence of fatal pneumonia with other causes of death in LT patients, data showed an almost homogenous rate. No COVID‐19‐associated pneumonia was observed

**Figure 3:**
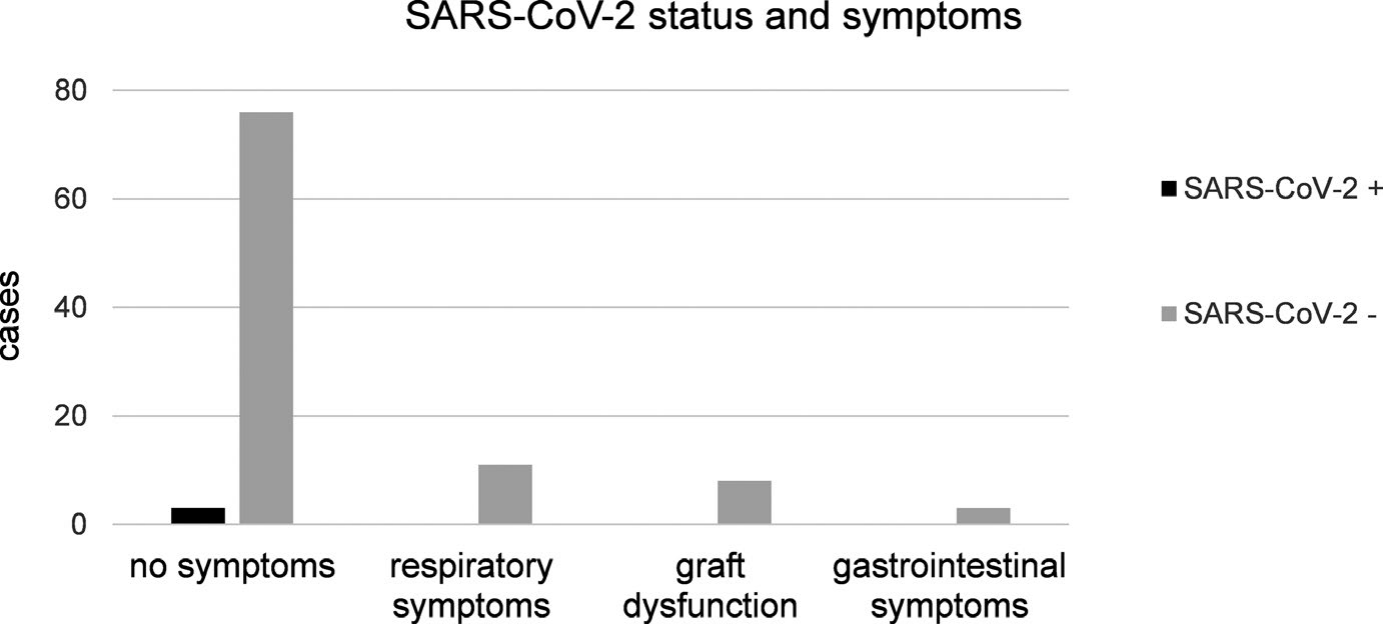
Symptoms or laboratory abnormalities at time of nasopharyngeal swab. In n = 101 patients, a nasopharyngeal swab was performed for detection of SARS‐CoV‐2 infection. Only n = 3 tested to be positive, and all were asymptomatic. Patients with respiratory symptoms either showed only mild symptoms or suffered from community aquired pneumonia. In cases with deterioration of liver function, no association of SARS‐CoV‐2 infection was found

N = 79 (78.2%) patients were completely asymptomatic, and 11 (10.9%) patients had respiratory symptoms, while eight (7.9%) were in a diagnostic work‐up of graft function disturbances and three (3.0%) had unspecific gastrointestinal complaints. SARS‐CoV‐2 PCR was positive in three asymptomatic patients, while it was negative in 98 patients including all 11 patients with reported respiratory symptoms of varying severity. All infections were reported to the Robert‐Koch Institute, the government's central scientific institution, responsible for surveillance of infectious diseases. There were three cases of COVID‐19‐negative bacterial pneumonia with confirmed radiological infiltrates Figure 2. Two patients were treated with antibiotics in the outpatient setting, and the other patient died following maximal treatment of exacerbated chronic obstructive pulmonary disease being tested negative for SARS‐CoV‐2. No other LT patient was diagnosed with SARS‐CoV‐2 infection at Charité Universitätsmedizin Berlin. The remaining eight patients presented only mild respiratory symptoms (coughing, mild stress dyspnea) without alteration of laboratory findings or impaired oxygenation, not requiring any further diagnostic steps. Observatory control after 1‐2 weeks showed no exacerbation of symptoms with complete spontaneous resolvement in n = 6 and decreasing symptoms in n = 2, and thus, diagnosis of unspecific upper respiratory tract infection was made. 50% (n = 11) of all symptomatic patients including non‐respiratory symptoms presented the usual spectrum of all clinical questions that arise in long‐term LT patients requiring answers in spite of the current pandemic. Moreover, no SARS‐CoV‐2‐associated disease was observed. Furthermore, patients with biochemical abnormalities regarding their graft function were all negative for SARS‐CoV‐2. There was no association between positive results of nasopharyngeal swabs for SARS‐CoV‐2 and symptoms in our patients (*P* = .835) as displayed in Figure 3.

**Figure 4:**
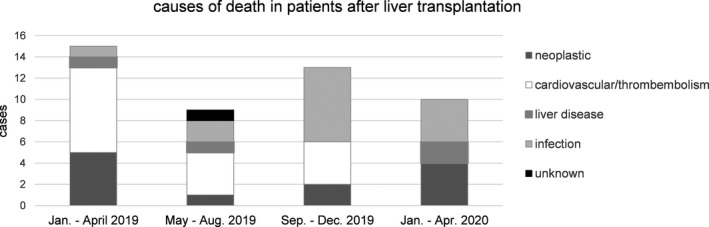
Post‐transplant mortality for 2019 including first 4 mo of 2020.Mortality of liver transplant patients was analyzed for cause of death. No increase in mortality in the first 4 mo of 2020 was noted

Moreover, no increase in the overall mortality of the entire cohort (n = 1500) was observed. In 2019, 37 patients died excluding early mortality (<6 months after LT), thus delivering a monthly rate of 0.21%. Within the first 4 months of 2020 during the SARS‐CoV‐2 pandemic, the number of deaths was 10, resulting in an identical monthly mortality rate of 0.22% as displayed in Figure 4. Of note, the outpatient clinic is in a steady state regarding the rate of new transplantations and mortality.

The first patient to test positive for SARS‐CoV‐2 was a 45‐year‐old male, 7 months after LT for hepatitis C (HCV)‐associated cirrhosis. Apart from active nicotine consumption, no other relevant comorbidities were known. Immunosuppressive regimen consisted of monotherapy with tacrolimus 4 mg/day with a trough level of 5,6 ng/mL. The recurrent HCV infection (genotype 3) was successfully treated with sofosbuvir/velpatasvir/voxilaprevir in a standard dose for 12 weeks. The patient had no respiratory or gastrointestinal symptoms and showed none in the 14‐day follow‐up. Home quarantine was imposed in accordance with current national guidelines.

The second patient was a 77‐year‐old woman 21 years after LT for primary biliary cholangitis. She was in a good shape and suffered from mild hypertension. Previously diagnosed bronchial carcinoma was treated by lung resection without chemotherapy 1 year before. The immunosuppression was mTor‐based (everolimus: trough level 5,6 ng/mL) in combination with low‐dose tacrolimus (trough level 1,5 ng/mL). However, no symptoms were evident at the positive swap, and the patient stayed asymptomatic during the 14 days of quarantine.

The last asymptomatic patient, who tested positive for SARS‐CoV‐2 in our cohort, was a 70‐year‐old male who underwent LT 12 years ago for HCV cirrhosis. Recurrent HCV (genotype 1a) was successfully treated with intravenous silibinin 10 years ago.^17^ There was no relevant report of comorbidities, and immunosuppression was stopped in an individual decision 2 months ago. The patient remained asymptomatic during the course of the 2‐week quarantine.

All positive SARS‐CoV‐2‐positive patients maintained a good and stable graft function without signs of inflammation assessed by laboratory (INR, bilirubin, and aminotransferases).

## 3 DISCUSSION

The results of the present study demonstrate that SARS‐CoV‐2 infection was a rare phenomenon in liver transplant patients in Berlin during the shutdown between March and April 2020. The few patients with a positive result from nasopharyngeal swab were completely asymptomatic regarding respiratory aspects of the potential disease. Our data are in accordance with D´Antiga et al stating that during the previous pandemics with SARS and MERS, no significant increase in the infection rate, severity of the disease, and mortality was associated with previous coronaviruses in immunosuppressed patients.^8^ The experience of three healthy pediatric liver transplant recipients tested positive for SARS‐CoV‐2 without any respiratory symptoms confirms our observation.

The backbone of modern immunosuppression is calcineurin inhibitors that block the activity of T‐lymphocytes and in combination with MMF also the activity of B cells thus hindering the inflammatory cascade directed toward the graft and other antigens. It has been discussed that this artificial imbalance in terms of rejection prophylaxis may result in an advantage by avoiding overwhelming immune reaction as cytokine storm, being primarily crucial for patients with ARDS. Immunosuppression may attenuate the innate immune reaction thus paradoxically being protective in this situation and avoid tissue damage.^18^

Interestingly, our asymptomatic graft recipients, though in a limited number, represented three common situations after LT in a long‐term follow‐up. The first patient transplanted 7 months ago was asymptomatic on CNI‐based immunosuppression. Our second patient was on a standard mTor‐based immunosuppression because of the successfully treated pulmonary neoplasm 21 years after LT for PBC, and our third patient was in a weaning program from tacrolimus‐ and MMF‐containing immunosuppression for 2 months. Of course, the number of patients is low. However, the presented cohort of 101 LT patients proves to belong to a high‐risk group of patients, comprising advanced age and a relevant number of comorbidities.

On the other hand, Bhoori et al^19^ reported on three long‐term LT patients who succumbed to severe SARS‐CoV‐2 infection being older than 65 years, male and suffering from metabolic syndrome, thus confirming the risk factors for the severity of the disease. However, no epidemiologic conclusion can be drawn from these series and well‐organized registries are therefore indispensable. At this point in time, the prevalence of SARS‐CoV‐2 infections in western countries can only be estimated. According to the German Robert‐Koch‐Institute, about 17% of SARS‐CoV‐2‐infected patients required hospitalization and 3% developed pneumonia. While death rate is stated to be 3.3% of all registered cases, median age of patients dying from COVID‐19 was 82 years in Germany.^20^ In Berlin, the prevalence of SARS‐CoV‐2 infection is reported to be 170/100 000 but the number of unrecorded cases is estimated to be 10‐20 times higher,^20^ thus resulting in an approximation of 1%‐4%. Interestingly, exactly this prevalence was found in our study.

The reported mortality rate of the present analysis as demonstrated through the last year up to 2020 into the SARS‐CoV2 pandemic is constant without change in 1500 living LT patients and does not show any significant changes. There was one COVID‐19‐negative death in March because of a severe chronic obstructive lung disease. Thus, the data collected in our cohort do not primarily support the view of LT patients being at high risk for COVID‐19, especially regarding the extent of comorbidities. A possible reason for this might be the fact that most of our transplanted patients are disciplined, well educated and consequently aware of their vulnerable status under immunosuppression. Also, immunosuppression in LT‐patients in general is lower compared to other solid organ transplants.^21^ For example, only 3% received immunosuppression with corticosteroids in our collective. Still, it is possible that LT patients from our clinic were SARS‐CoV‐2 positive or diagnosed with COVID‐19 in another hospital or institution thus leading to an underestimation of prevalence. Due to excellent patients´ adherence and lifelong care in our outpatient clinic, we expect this bias to be negligible as on the contrary, patients frequently visit the outpatient clinic with various medical needs without regard of LT‐specific care. Also, due to its exploratory character, this study was not randomized.

We did not perform follow‐up swabs in patients without severe symptoms or clinical self‐resolving course in accordance with laboratory recommendations to generate capacities for testing of urgent cases. However, personnel was specifically trained to perform adequate nasopharyngeal swabs according to recommendations of the World Health Organization (WHO) by internal standard operating procedures (SOPs) and PCR is reported to have high sensitivity.^15^

Usual and frequent conditions such as acute rejection, cholangitis, ischemic‐type biliary lesions should deserve the same amount of attention due to their significance after LT with the need for treatment persisting with an unaltered prevalence despite a pandemic. These treatable pathologies should be first priority in endangered, high‐risk patients, and optimal treatment is required to affect the overall prognosis in a positive manner.

Despite from that, countermeasures that took place in this pandemic including reduction in social activity (“social distancing”), basic hygiene, and overall reasonable actions may very well contribute to the favorable results.^22^ However, even under these remarkable conditions, routine medical treatment practices and rationales of guidelines should be followed.

## 4 CONCLUSION

Respiratory complaints among stable liver transplant patients are common and were not associated with SARS‐CoV‐2 infection in the presented cohort. If preventive measures are consequently applied, LT patients do not seem to be at a higher risk for SARS‐CoV‐2 infection. The spectrum of mortality is normal in an institution with reduced activity during the shutdown in Germany. Therefore, telemedicine in the outpatient setting may help to keep patient contact to a minimum. However, standard care for patients who require immediate medical attention must be guaranteed.

## CONFLICT OF INTEREST

All authors declare no conflict of interest related to the presented work.

5

**Table 1:** Demographic data of 101 liver transplant patient

N = 101	
Median age at LT in years; (Min.‐Max.)	51.3 (3.3‐68.7)
Median age in April 2020 in years; (Min.‐Max.)	64.0 (19.8‐86.8)
Time since LT in years; (Min.‐Max.)	11.0 (0.07‐31.7)
Gender; n (%)	
Male	55 (54.5)
Female	46 (45.5)
Indication for LT; n (%)	
Alcohol	19 (18.8)
Viral (HBV and HCV)	32 (31.7)
Autoimmune (AIH, PBC, PSC)	17 (16.8)
NASH/ cryptogenic	7 (6.9)
Acute liver failure	11 (10.9)
All others	15 (14.9)
Immunosuppression; n(%)	
CNI mono	30 (29.7)
CNI + MMF	38 (37.6)
CNI + mTOR	12 (11.9)
None	9 (8.9)
Others	12 (11.9)
Comorbidities; n (%) (at least 1 of 8)	82 (81.2)
Obesity; BMI=>30 kg/cm^2^	16 (15.8)
Cardiovascular pathologies;	52 (51.5)
Arterial hypertension	43 (42.6)
Coronary heart disease	18 (17.8)
Pulmonary disease	27 (26.7)
Active smoke	14 (13.9)
Diabetes mellitus	25 (24.8)
Neoplastic disease	16 (15.8)
IBD	10 (9.9)
CKD higher than IIIb (eGFR <45 mL/min)	17 (16.8)

Note

N = 101 patients that visited our outpatient clinic were tested for SARS‐CoV‐2 infection via nasopharyngeal swab. Of note, only three patients received an immunosuppressive regimen with combination therapy of corticosteroids.

Abbreviations: CKD, chronic kidney disease; CNI, calcineurin inhibitor; HBV/HCV, hepatitis B/C‐associated liver disease; IBD, inflammatory bowel disease; MMF, mycophenolate mofetil; mTor, mammalian Target of Rapamycin.
